# A new method for insertion of long intestinal tube for small bowel obstruction: Nonendoscopic over-the-wire method via short nasogastric tube

**DOI:** 10.1097/MD.0000000000005449

**Published:** 2016-11-28

**Authors:** Kazuma Sekiba, Tomoya Ohmae, Nariaki Odawara, Makoto Moriyama, Sachiko Kanai, Mayo Tsuboi, Tomotaka Saito, Koji Uchino, Masatoshi Akamatsu, Makoto Okamoto

**Affiliations:** Department of Gastroenterology, JR Tokyo General Hospital, Tokyo, Japan.

**Keywords:** long intestinal tube, NEWSt, short nasogastric tube, small bowel obstruction

## Abstract

It is often difficult to insert a long intestinal tube (LT) in patients with small bowel obstruction (SBO). We developed a novel technique for inserting an LT without endoscopy called nonendoscopic over-the-wire method via short nasogastric tube (NEWSt). We evaluated the efficacy and safety of NEWSt.

We performed a retrospective study of patients who underwent LT insertion for SBO without any indications of strangulation with either NEWSt (n = 16) or endoscopy (n = 17) between November 2011 and February 2015 at our hospital. Univariate analysis was used to assess the success rate of LT placement beyond the duodenojejunal flexure, time required for the procedure, clinical outcomes, and adverse events.

The success rate was 100% in both groups. Procedure time was numerically, but not statistically, shorter in the NEWSt group compared with the endoscopy group (24 ± 13 vs 30 ± 13 min; *P* = 0.174). There were no statistically significant differences between the 2 groups in terms of surgery rate (31% vs 12%; *P* = 0.225), fasting period (11.3 ± 6.3 vs 9.9 ± 4.5 days; *P* = 0.482), hospital stay (26.4 ± 22.1 vs 18.7 ± 7.0 days; *P* = 0.194), and recurrence rate (19% vs 24%; *P* = 1.0). No serious adverse event was observed in the NEWSt group, whereas serious aspiration pneumonia was observed in 2 patients after LT insertion in the endoscopy group.

Without endoscopy, NEWSt enabled the high success rate and the short procedure time for the LT insertion. Prospective, randomized controlled trials are needed.

## 1 Introduction

Gastrointestinal decompression is the most effective therapy for the patients with acute small bowel obstruction (SBO) without any indications of strangulation.^[1–4]^ There are 2 ways of bowel decompression, with insertion of a short nasogastric tube (NGT) or of a long intestinal tube (LT). NGT is easier to insert than LT, but it is less effective for suctioning the distal intestine. A randomized trial indicated that there was no advantage of decompression by LT compared with such treatment by NGT^[5]^; however, in that study, the placement of LT beyond the duodenojejunal flexure was not confirmed using fluoroscopy. In addition, there have been reports from many countries describing the usefulness of LT decompression for the management of SBO.^[1–4,6–8]^ Therefore, if the clinical course after NGT insertion is not satisfactory, we have to consider inserting the LT instead.

When exchanging an NGT to an LT, the NGT is usually completely withdrawn at first, followed by reinsertion of the LT. However, LT insertion is often a difficult and time-consuming procedure and causes severe patient distress. Currently, transnasal ultrathin endoscopy-assisted LT insertion has been established.^[9–17]^ Several studies have revealed that endoscopic insertion is superior to conventional fluoroscopic insertion in terms of the success rate of LT placement beyond the duodenojejunal flexure and the time required for the procedure.^[15–17]^ However, endoscopic insertion always requires a thin-caliber endoscope and an endoscopist. Therefore, the procedure cannot be performed at all hospitals. Moreover, aspiration pneumonia may sometimes occur after endoscope insertion.

Therefore, we developed a novel technique called nonendoscopic over-the-wire method via short nasogastric tube (NEWSt) for easy and safe LT insertion without endoscopy. In this study, we assessed the usefulness and safety of NEWSt for patients with SBO.

## 2 Methods

### 2.1 Patients

In all, 35 consecutive patients who underwent LT insertion for SBO without any indications of strangulation at the JR Tokyo General Hospital between November 2011 and February 2015 were included in this retrospective study. Computed tomography (CT) scan of abdomen and pelvis was performed in all the patients on admission to aid the diagnosis, etiology, and severity of SBO. Generalized peritonitis on physical examination or other evidence of clinical deterioration such as fever, leukocytosis, tachycardia, metabolic acidosis, massive ascites, and continuous pain were considered as the indication of strangulation. Patients with any of the following characteristics were excluded: prior total gastrectomy, and/or existence of obstructive tumor. Patients were assigned to either of the following groups based on the method of LT insertion: NEWSt or transnasal ultrathin endoscopy-assisted.

### 2.2 Instruments and procedures

All procedures were performed in the x-ray suite of our hospital. The method of LT insertion was determined by the attending physician of each patient.

#### 2.2.1 NEWSt

**Figure 1:**
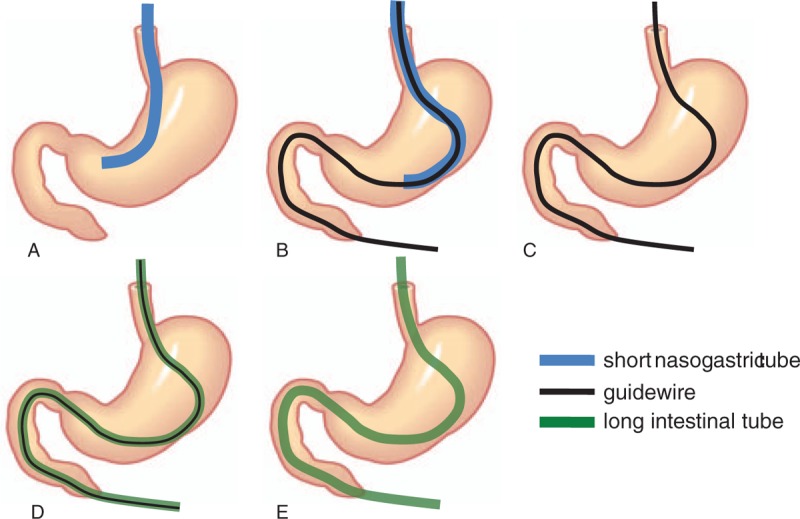
Procedure of LT insertion using nonendoscopic over-the-wire method via short nasogastric tube (NEWSt). (A) A short nasogastric tube (NGT) is placed in the stomach before NEWSt. (B) A Dennis guidewire (1.32 mm, 500 cm long; Covidien, Tokyo, Japan) is inserted via NGT as distally as possible beyond the duodenojejunal flexure under fluoroscopy guidance. (C) The NGT is withdrawn while the guidewire is kept in place. (D) A hydrophilic long tube (16 Fr, 300 cm; Argyle Super Dennis Tube; Covidien, Tokyo, Japan) is inserted along the guidewire. (E) The guidewire is withdrawn. LT = long intestinal tube.

**Figure 2:**
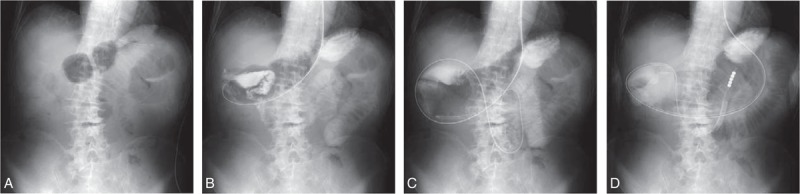
Radiographs of NEWSt. (A) A short nasogastric tube (NGT) is placed in the stomach. (B) A Dennis guidewire (1.32 mm, 500 cm long; Covidien, Tokyo, Japan) is inserted via NGT. (C) The guidewire is advanced beyond the duodenojejunal flexure. (D) After NGT is withdrawn, a hydrophilic long tube (16 Fr, 300 cm; Argyle Super Dennis Tube; Covidien, Tokyo, Japan) is inserted along the guidewire, which is withdrawn. NEWSt = nonendoscopic over-the-wire method via short nasogastric tube.

For local anesthesia, 8% lidocaine was sprayed in the pharynx and a small amount of 2% lidocaine jelly was applied to the nostrils. A 1.32-mm wide, 500-cm long Dennis guidewire (Covidien, Tokyo, Japan) was inserted via NGT with the patient in the supine position. The guidewire was advanced beyond the duodenojejunal flexure under fluoroscopy guidance. NGT was then withdrawn while the guidewire was kept in place. Subsequently, a 16-Fr, 300-cm hydrophilic long tube (Argyle Super Dennis Tube; Covidien, Tokyo, Japan) was gently inserted along the guidewire. The tube was advanced as distally as possible beyond the duodenojejunal flexure. Finally, the positioning balloon was inflated, and the guidewire was withdrawn (Figs. 1 and 2).

#### 2.2.2 Endoscopy-assisted

For local anesthesia, application of a drop of naphazolone nitrate and 4% lidocaine spray on the nostrils were performed. A thin-caliber endoscope (GIF-XP260N; Olympus, Tokyo, Japan) was transnasally inserted with the patient in the semiprone position. The scope was advanced to the duodenum. The guidewire was inserted via the working channel of the endoscope into the small bowel and beyond the duodenojejunal flexure under fluoroscopy guidance. The endoscope was then withdrawn while the guidewire was kept in place. Afterward, the tube was indwelled in a similar way as described in NEWSt.

### 2.3 Baseline characteristics

Data including age, sex, body mass index, and previous abdominal surgery were obtained from the medical records.

### 2.4 Study endpoints

The primary outcomes of interest were success rate and procedure time, and the secondary outcomes were the clinical outcomes including surgery rate, fasting period, hospital stay, and recurrence rate, and complications. Successful intubation was defined as LT insertion beyond the duodenojejunal flexure. Procedure time was determined from guidewire insertion in NEWSt group and from endoscope insertion in the endoscopy group. Surgery rate was the percentage of patients who needed surgery for SBO treatment, and recurrence rate was that of patients who had SBO relapse after withdrawal of the LT. Fasting period was calculated as the interval between admission and resumption of oral intake.

A diagnosis of aspiration pneumonia as a complication required the presence of new radiographic infiltrate/s, an aspiration event during LT insertion, and at least 1 of the following: symptoms of infection, such as fever (>38.0°C), productive cough, purulent sputum from the lower respiratory tract, or chest pain; or white blood cell count >10,000/μL and/or elevated C-reactive protein >0.03 mg/dL. The I-ROAD classification of the revised Japanese Respiratory Society guidelines on healthcare-associated pneumonia^[18]^ was used for evaluating the severity of pneumonia.

### 2.5 Statistical analyses

All statistical analyses were conducted using the R program version 3.1.2 (R Development Core Team 2014, The R Foundation for Statistical Computing; Vienna, Austria). Continuous variables were reported as mean ± standard deviation (SD), and categorical variables were reported as frequency and percentage. Welch *t* test was used for group comparisons of continuous variables. Fisher exact test was used for group comparisons of categorical variables. *P* values of <0.05 were considered as statistically significant.

### 2.6 Ethics

This study was conducted in accordance with the Declaration of Helsinki and the ethical guidelines for epidemiological research developed by the Ministry of Education, Culture, Sports, Science and Technology, and the Ministry of Health, Labor and Welfare, Japan. Written informed consents were obtained from all the patients or their relatives before performing LT insertion. The study design was approved by the ethics committee of the author's institution (registration no. H27–16).

## 3 Results

### 3.1 Patients

**Figure 3:**
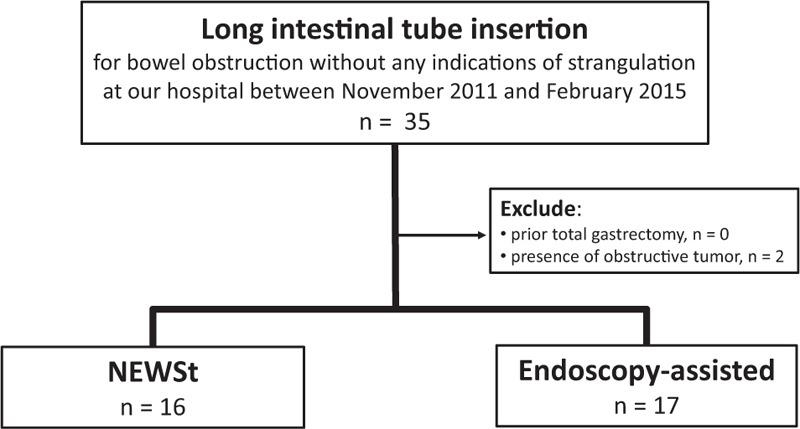
Flowchart of subject inclusion in the study. Between November 2011 and February 2015, 35 patients underwent long intestinal tube insertion for small bowel obstruction without any signs of strangulation. Two met the exclusion criteria. Sixteen patients underwent NEWSt; 17 patients underwent endoscopy-assisted insertion. NEWSt = nonendoscopic over-the-wire method via short nasogastric tube.

In all, 33 patients met the inclusion criteria: 16 underwent NEWSt and 17 underwent the endoscopy-assisted method (Fig. 3). The 2 patients who were excluded from this study due to the obstructive tumor were of pancreas cancer and cecum cancer. Both the tumors were massive enough to be detected by CT scanning on admission. Additionally, these were confirmed by autopsy and surgery, respectively. NEWSt was performed within 24 hours after NGT insertion in 13 patients (81%). The 2 groups were similar in baseline characteristics (Table 1). In the endoscopy group, 3 patients had a history of partial gastrectomy with Billroth-I method.

**Table 1:**
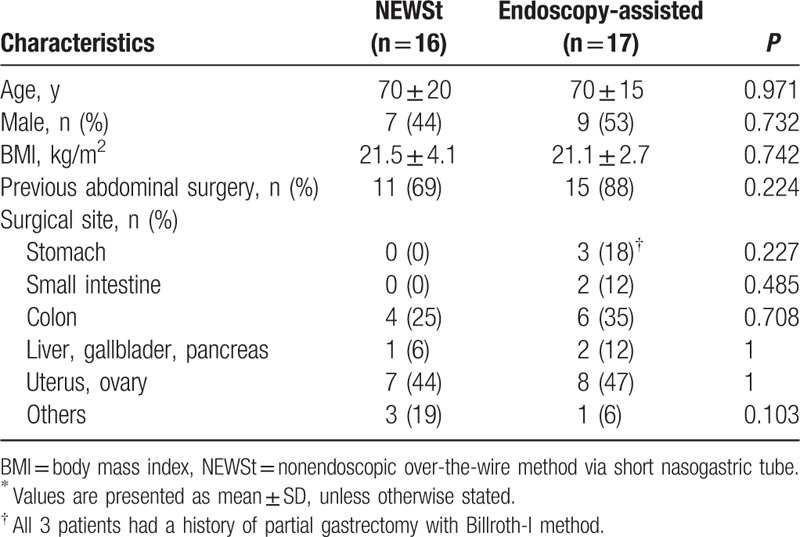
Baseline characteristics of the study population^∗^.

### 3.2 Success rate and procedure time

Success rate was 100% in both groups (*P* = 1.0). The procedure time was numerically, but not statistically, shorter in the NEWSt group compared with the endoscopy group (24 ± 13 vs 30 ± 13 min; *P* = 0.174) (Table 2).

**Table 2:**
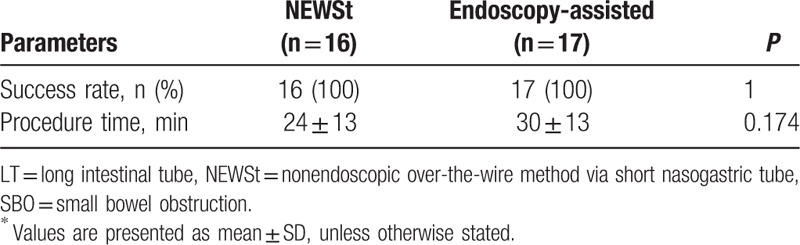
Comparison of procedures for LT insertion for SBO^∗^.

### 3.3 Clinical outcome and complications

There were no statistically significant differences between the 2 groups in terms of surgery rate (31% vs 12%; *P* = 0.225), fasting period (11.3 ± 6.3 vs 9.9 ± 4.5 days; *P* = 0.482), hospital stay (26.4 ± 22.1 vs 18.7 ± 7.0 days; *P* = 0.194), and recurrence rate (19% vs 24%; *P* = 1.0) (Table 3).

**Table 3:**
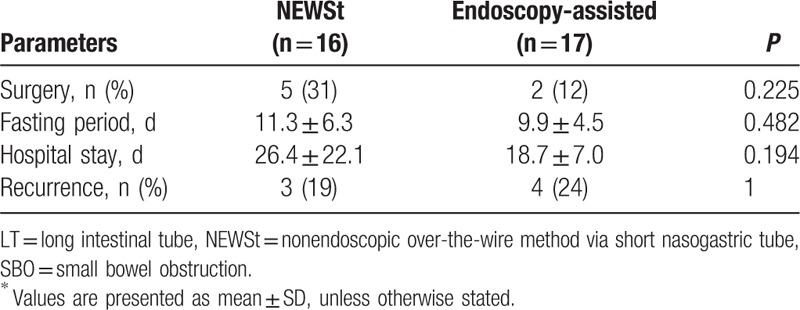
Clinical outcomes of patients who underwent LT insertion for SBO^∗^.

In NEWSt group, 1 patient had epistaxis due to contact with the guidewire; in this case, bleeding was minimal and was spontaneously resolved. No serious adverse event was observed in the NEWSt group, whereas moderately severe aspiration pneumonia after LT insertion was observed in 2 patients in the endoscopy group (Table 4).

**Table 4:**
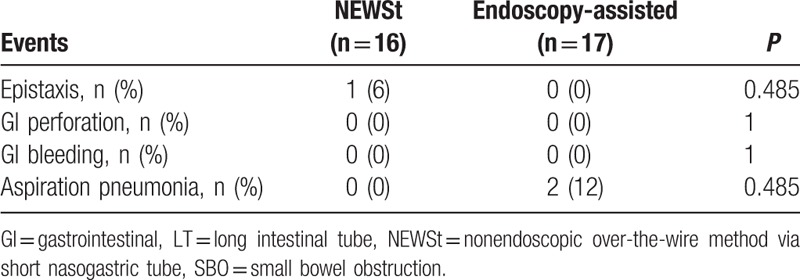
Adverse events in patients who underwent LT insertion for SBO.

## 4 Discussion

In this study, we observed that NEWSt provided a high success rate of LT insertion without endoscopy. Compared with endoscopy-assisted insertion, the procedure time tended to be shorter. In addition, the NEWSt group was similar to the endoscopy group in clinical outcomes; however, although the NEWSt group had no serious adverse events, the endoscopy group comprised 2 patients who developed serious aspiration pneumonia.

Former studies reported that endoscopy-assisted insertion was superior to conventional fluoroscopic insertion in terms of the success rate. It was reported to be about 88% through conventional fluoroscopic insertion, and 100% through endoscopic insertion.^[15–17]^ In the current study, NEWSt successfully placed the LTs for SBO in 100% (16/16) cases. Leading the guidewire (or endoscope) to the duodenum is difficult if the stomach is expanded due to SBO. This would be a main cause of LT insertion fails. Placement of the NGT in the stomach before LT insertion may solve such a problem by reducing gastric contents. Therefore, the use of a previously inserted NGT during NEWSt facilitated smooth LT insertion without the need for endoscopy, but with a similar high success rate.

Procedure time of NEWSt tended to be shorter than that of endoscopy-assisted insertion. The mean procedure time of LT insertion was reported 26 to 40 minutes through conventional fluoroscopic insertion and 16 to 19 minutes by endoscopic insertion.^[15–17]^ In this study, NEWSt successfully placed LT in 24 ± 13 minutes. Taking the most time in LT insertion is to pass through the pylorus. As mentioned in the former paragraph, NEWSt could make it easier by using the NGT. Although passing through the pharynx can be another time-consuming process of LT insertion, it did not matter for NEWSt because the NGT had already been placed.

The NEWSt group was similar to the endoscopy group in clinical outcomes, although the surgery rate, fasting period, and hospital stay tended to be longer in the NEWSt group. In the present study, NEWSt was performed only for the patients whose clinical course after NGT insertion was not satisfactory. Thus, it was possible that the NEWSt group potentially had more patients who needed surgery than the endoscopy group. In fact, 21 patients with SBO were treated only through the NGT in the study period. Then, the initially inserting NGT group, which was composed of the patients treated only through the NGT and through NEWSt, was almost same to the endoscopy group in the surgery rate (14% vs 12%; *P* = 1.0). There were also no differences between the 2 groups in the fasting period (7.5 ± 5.5 vs 9.9 ± 4.5 days; *P* = 0.089) and hospital day (17.1 ± 16.9 vs 18.7 ± 7.0 days; *P* = 0.631).

No reports have shown whether regurgitation and air insufflation during endoscopy aggravates the condition of patients with SBO. Nevertheless, the absence of aspiration pneumonia in patients who underwent NEWSt may have been due to the indwelling NGT that decompressed stomach before LT insertion.

The advantages of NEWSt arise from the unnecessity of the endoscopy. NEWSt can be performed with the use of standard techniques and equipments of the conventional fluoroscopic LT insertion. In addition, the endoscopists do not need to be called when performing NEWSt.

The main limitation of our study was its retrospective design, which may have contributed some selection bias. In addition, the criteria for performing NEWSt and endoscopy-assisted LT insertion were not standardized in this study. This design is limited also in that it does not allow direct comparison with other forms of therapy; such as the conventional fluoroscopic insertion and the endoscopic insertion followed by NGT insertion. However, the advantage of NEWSt, not requiring endoscopy, was never impaired by such limitations.

In conclusion, this retrospective study showed that NEWSt, a novel method for LT insertion, provided high success rate and short procedure time without any severe complications. Prospective, randomized controlled trials are needed to confirm these results.

## References

[1] Wangsteen OH Paine JR Treatment of intestinal obstruction by suction with a duodenal tube. JAMA 1993;101:1532–9.

[2] Wangsteen OH Historical aspects of the management of acute intestinal obstruction. Surgery 1969;65:363–83. 4885351

[3] Wangsteen OH Understanding the bowel obstruction problem. Am J Surg 1978;135:131–49. 343612 10.1016/0002-9610(78)90087-9

[4] Hofstetter SR Acute adhesive obstruction of the small intestine. Sure Gynecol Obstet 1981;152:141–4. 7209754

[5] Fleshner PR Siegman MG Slaster GL A prospective, randomized trial of short versus long tubes in adhesive small-bowel obstruction. Am J Surg 1995;170:366–70. 7573730 10.1016/s0002-9610(99)80305-5

[6] Gowen GF Long tube decompression is successful in 90% of patients with adhesive small bowel obstruction. Am J Surg 2002;185:512–5. 10.1016/s0002-9610(03)00074-6 12781876

[7] Tanaka S Yamamoto T Kubota D Predictive factors for surgical indication in adhesive small bowel obstruction. Am J Surg 2008;196:23–7. 18367141 10.1016/j.amjsurg.2007.05.048

[8] Chen XL Ji F Lin Q A prospective randomized trial of transnasal ileum tube vs nasogastric tube for adhesive small bowel obstruction. World J Gastroenterol 2012;18:1968–74. 22563179 10.3748/wjg.v18.i16.1968 PMC3337574

[9] Keller RT A technique of intestinal intubation with fiberoptic endoscope. Gut 1973;14:143–4. 4633161 10.1136/gut.14.2.143 PMC1412561

[10] Chung RSK Denbesten L Improved technique for placement of intestinal feeding tube with the fiberoptic endoscope. Gut 1976;17:264–6. 817986 10.1136/gut.17.4.264 PMC1411093

[11] Johnson FW Goodale RL Leonard AS Rapid long tube intubation of the jejunum by a new endoscopic device. Am J Surg 1976;131:91–3. 1247160 10.1016/0002-9610(76)90427-x

[12] Morrissey JF Douglas DD An endoscopic method for the rapid passage of tubes into the small intestine. Gastroenterol Endosc 1978;20:1247–9.

[13] Kawamura R Okabe M Misumi A Rapid long tube intubation of the jejunum-an improved technique. Jpn J Surg 1984;14:299–304. 6492503 10.1007/BF02469645

[14] Endo H Inamori M Murakami T Usefulness of transnasal ultrathin endoscopy for the placement of a post pyloric decompression tube. Digestion 2007;75:181. 17823509 10.1159/000107937

[15] Sato R Watari J Tanabe H Transnasal ultrathin endoscopy for placement of a long intestinal tube in patients with intestinal obstruction. Gastrointest Endosc 2008;67:953–7. 18440385 10.1016/j.gie.2008.01.043

[16] Kanno Y Hirasawa D Fujita N Long-tube insertion with the ropeway method facilitated by a guidewire placed by transnasal ultrathin endoscopy for bowel obstruction: a prospective, randomized, controlled trial. Gastrointest Endosc 2009;69:1363–8. 19481656 10.1016/j.gie.2009.01.044

[17] Guo SB Duan ZJ Decompression of the small bowel by endoscopic long-tube placement. World J Gastroenterol 2012;18:1822–6. 22553408 10.3748/wjg.v18.i15.1822 PMC3332297

[18] Kohno S Imamura Y Shindo Y Clinical practice guidelines for nursing- and healthcare- associated pneumonia (NHCAP) [complete translation]. Respire Investig 2013;51:103–26. 10.1016/j.resinv.2012.11.001 23790739

